# Development of a Two-Dimensional Liquid Chromatographic Method for Analysis of Urea Cycle Amino Acids

**DOI:** 10.3390/molecules29030700

**Published:** 2024-02-02

**Authors:** Yuko Sumida, Makoto Tsunoda

**Affiliations:** Graduate School of Pharmaceutical Sciences, University of Tokyo, Tokyo 113-0033, Japan

**Keywords:** arginine, ornithine, citrulline, argininosuccinate, fluorescence

## Abstract

The urea cycle has been found to be closely associated with certain types of cancers and other diseases such as cardiovascular disease and chronic kidney disease. An analytical method for the precise quantification of urea cycle amino acids (arginine, ornithine, citrulline, and argininosuccinate) by off-line two-dimensional liquid chromatography (2D-LC) combined with fluorescence-based detection was developed. Before analysis, the amino acids were derivatised with 4-fluoro-7-nitro-2,1,3-benzoxadiazole (NBD-F) to obtain NBD-amino acids. The first dimension involved the reversed-phase separation, in which NBD derivatives of urea cycle amino acids were completely separated from each other and mostly separated from the 18 NBD-proteinogenic amino acids. The samples were eluted with stepwise gradient using 0.02% trifluoroacetic acid in water–acetonitrile as the mobile phase. In the second dimension, an amino column was used for the separation of NBD-ornithine, -citrulline, and -argininosuccinate, while a sulfonic acid column was used to separate NBD-arginine. The developed 2D-LC system was used to analyse human plasma samples. The fractions of NBD-urea cycle amino acids obtained in the first dimension were collected manually and introduced into the second dimension. By choosing appropriate mobile phases for the second dimension, each NBD-urea cycle amino acid eluted in the first dimension was well separated from the other proteinogenic amino acids and interference from endogenous substance. This could not be achieved in the first dimension. The urea cycle amino acids in human plasma sample were quantified, and the method was well validated. The calibration curves for each NBD-urea cycle amino acid showed good linearity from 3 (ASA) or 15 (Orn, Cit, and Arg) to 600 nM, with correlation coefficients higher than 0.9969. The intraday and interday precisions were less than 7.9% and 15%, respectively. The 2D-LC system is expected to be useful for understanding the involvement of the urea cycle in disease progression.

## 1. Introduction

**Figure 1:**
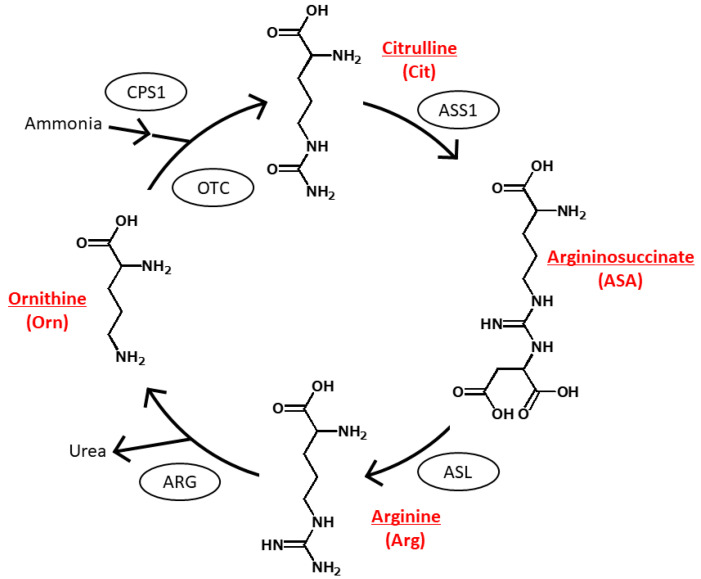
Urea cycle and chemical structures of urea cycle amino acids. The encircled compounds are the enzymes involved in the respective steps. CPS1, carbamoyl phosphate synthase 1; OTC, ornithine transcarbamylase; ASS1, argininosuccinate synthase 1; ASL, argininosuccinate lyase; and ARG, arginase.

The urea cycle is an essential biochemical pathway in mammals for the conversion of excess nitrogen from toxic ammonia and other metabolites into urea, which is ultimately excreted by the mammals. The significance of the urea cycle extends beyond ammonia detoxification, encompassing various metabolic processes, including the synthesis and catabolism of arginine (Arg) and its interconnection with the tricarboxylic acid cycle. Figure 1 shows the four amino acids, namely Arg, ornithine (Orn), citrulline (Cit), and argininosuccinate (ASA), that are involved in the urea cycle and the five enzymes, namely carbamoyl phosphate synthase 1 (CPS1), ornithine transcarbamylase (OTC), argininosuccinate synthase 1 (ASS1), argininosuccinate lyase (ASL), and arginase (ARG), that catalyses various steps of the urea cycle, orchestrating the conversion of nitrogenous waste into urea for safe excretion.

However, disruptions in the urea cycle can lead to urea cycle disorders, which manifest as deviations in the ammonia detoxification pathway [1,2,3,4]. These disorders are often attributed to partial or complete deficiencies in the enzymes and transporters associated with the urea cycle. Such deficiencies can result in the accumulation of toxic ammonia, leading to a range of clinical symptoms and complications. Understanding the intricacies of the urea cycle, its molecular components, and the enzymes involved is crucial for unravelling the mechanisms underlying urea cycle disorders. Advances in this field not only contribute to clinical diagnostics and treatment strategies but also shed light on broader aspects of nitrogen metabolism in mammalian physiology.

In recent investigations, alterations in the expression of the enzymes related to the urea cycle have been identified in the context of cancer [5,6]. Notably, ASS1 exhibits overexpression in various cancer types, contributing to the enhanced proliferation of human colorectal cancer cells [7,8]. This upregulated expression of ASS1 in specific cancers, including colorectal cancer, suggests its potential role as a target for therapeutic interventions. Conversely, in certain cancers such as pancreatic cancer and hepatocellular carcinoma, ASS1 is found to be downregulated, indicating diverse regulatory mechanisms in different malignancies [9,10,11]. Furthermore, the association between the decreased expression of ASL and poor survival outcomes has been observed in hepatocellular carcinoma patients [12]. This emphasizes the complex interplay of urea cycle enzymes in the context of cancer progression and prognosis.

Beyond cancer research, recent studies have unveiled the diagnostic potential of urea cycle profiles in various diseases [13,14,15,16,17]. Clinical applications extend to chronic kidney disease, chronic fatigue syndrome, and cardiovascular disease, where abnormalities in urea cycle amino acids can serve as indicative markers. These findings underscore the broader significance of understanding urea cycle dynamics not only in cancer but also in diverse pathological conditions. Given the multifaceted role of urea cycle amino acids, their measurement in biofluids emerges as a crucial aspect for clinical assessments. Establishing comprehensive profiles of urea cycle components in biofluids holds promise for advancing diagnostic methodologies and gaining insights into the pathophysiology of a spectrum of diseases, thereby contributing to personalized medicine approaches.

In the realm of amino acid analysis, various analytical methodologies have been devised over the years to determine individual or subsets of the four crucial urea cycle amino acids [18,19,20,21,22,23]. A notable example comes from the work of Lai et al., who introduced a validated method leveraging hydrophilic interaction chromatography and tandem mass spectrometry. This method successfully enabled the simultaneous quantification of arginine (Arg), ornithine (Orn), and citrulline (Cit) in human plasma. However, despite such advancements, there exists a discernible gap in methodologies that allow for the concurrent quantification of all four urea cycle amino acids. Recognizing the pivotal role of the urea cycle in disease progression, the imperative arises to address this gap and establish robust methodologies that facilitate the simultaneous quantification of all four urea cycle amino acids. Such comprehensive quantification holds the key to unlocking a holistic understanding of the urea cycle’s intricate involvement in various diseases, potentially paving the way for more accurate diagnostics and targeted therapeutic interventions.

Given this context, the primary objective of the current study was to establish a technique that is both sensitive and accurate, facilitating the simultaneous determination of urea cycle amino acids in human plasma samples. As a result, an off-line two-dimensional liquid chromatography (2D-LC)-based method was meticulously developed and implemented. The methodology encompassed a series of systematic steps, commencing with the precolumn fluorescence derivatization of the target amino acids. Subsequently, the first dimension involved reversed-phase separation, enhancing the precision of amino acid isolation. Following this, the second dimension of the method comprised ion-exchange separation, facilitating the meticulous fractionation of each amino acid. This innovative approach not only allowed for enhanced sensitivity in detection but also ensured the accuracy required for simultaneous determination. By combining the advantages of 2D-LC and fluorescence derivatization, this method presents a robust platform for advancing the analytical capabilities in quantifying urea cycle amino acids in complex human plasma samples.

## 2. Results and Discussion

### 2.1. Optimization of the Separation of NBD-Urea Cycle Amino Acids on Reversed-Phase Column

To attain heightened sensitivity in the detection of the analytes, a crucial step involved the derivatization of amino acids utilizing a fluorescent reagent, namely NBD-F. The introduction of the NBD-F reagent, characterized by its fluorescent properties, served the dual purpose of rendering the amino acids amenable to fluorescence detection while ensuring their effective separation and retention within the chromatographic system. This derivatization strategy played a pivotal role in optimizing the analytical conditions, ultimately contributing to the method’s capacity for precise and sensitive quantification of amino acids in complex biological samples.

In the reversed-phase column, the three hydrophilic amino acids derivatised with NBD-F (i.e., NBD-Arg, NBD-Cit, and NBD-ASA) were separated from other NBD-proteinogenic hydrophilic amino acids (His, Asn, Gln, Ser, Asp, and Gly). Since we found that the NBD-proteinogenic amino acids could be successfully separated using citrate buffer–acetonitrile as the mobile phase [24], we speculated that the same mobile phase could also be used for the separation of the analytes in this study. However, under none of the conditions (pH or citrate concentrations) investigated, Arg was separated from the other amino acids or by-products of the NBD reaction. Hence, the mobile phase was changed to TFA and acetonitrile, which was already used for the separation of NBD-amino acids [25,26]. Although several conditions were tested, it was not possible to separate all the three NBD-urea cycle amino acids from the other NBD-hydrophilic amino acids under any condition. As the plasma sample could contain endogenous compounds other than the investigated amino acids, it seemed difficult to completely separate the NBD-urea cycle amino acids only using the reversed-phase column. This motivated the development of the 2D-LC system.

The TFA concentration in the mobile phase (water–acetonitrile, 80/20 (*v/v*)) was varied from 0.02% to 0.14%, to find an optimum condition for the separation of NBD-Arg, -Cit, and -ASA from each other. The resolution of each pair (NBD-ASA and NBD-Arg, NBD-Arg and NBD-Cit, and NBD-ASA and NBD-Cit) decreased with increasing TFA concentration, and 0.02% TFA was found to be the optimum condition. This concentration was used for further studies. 

Next, the separation of NBD-Orn from 18 other NBD-proteinogenic amino acids was examined. NBD-Orn has two NBD moieties because of the two amino groups in Orn and has a long retention on an ODS column. Under the optimised conditions as described in the experimental section, NBD-Orn was well resolved from the 18 NBD-proteinogenic amino acids upon stepwise gradient elution. 

**Figure 2:**
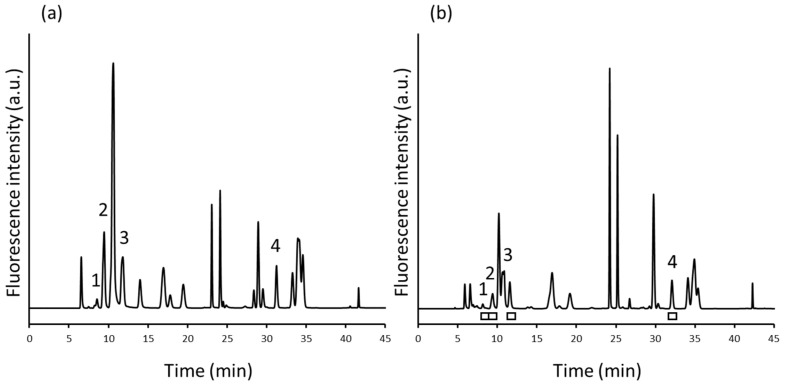
Reversed-phase separation of (**a**) NBD-derivatives of argininosuccinate (ASA), arginine (Arg), citrulline (Cit), ornithine (Orn), and 18 proteinogenic amino acid standards and (**b**) human plasma sample. Peaks: 1, NBD-ASA; 2: NBD-Arg; 3, NBD-Cit; 4, NBD-Orn. The fractions indicated by white bars were collected and transferred to the second dimension. Column: Inertsil ODS-4 (1.5 mm × 250 mm, 5 μm, GL Sciences, Tokyo, Japan). Mobile phases: (A) water–acetonitrile–TFA (80/20/0.02, *v/v/v*) and (B) water–acetonitrile–TFA (20/80/0.02, *v/v/v*). Stepwise elution: 0–15 min: 0% B, 15.01–35 min: 40% B, 35.01–45 min: 100% B.

Figure 2a shows the chromatogram of NBD-amino acids. NBD-urea cycle amino acids were completely separated from each other and mostly separated from the 18 NBD-proteinogenic amino acids.

### 2.2. Retention of NBD-Urea Cycle Amino Acids on Ion-Exchange Column

2D-LC can increase the resolution of the peaks and enhance the specificity of the method as compared with one-dimensional separation [27,28,29,30]. As mentioned before, this method was chosen because the separation of the NBD-urea cycle amino acids from the 18 NBD-proteinogenic amino acids on an ODS column was difficult. In 2D-LC, high orthogonality is important, and the mode of separation must be different from reversed-phase separation, which was used in the first dimension. Since NBD-amino acids have one or more free carboxylic acid groups, an amino column, which is capable of ion exchange and hydrophilic interactions, was chosen.

**Figure 3:**
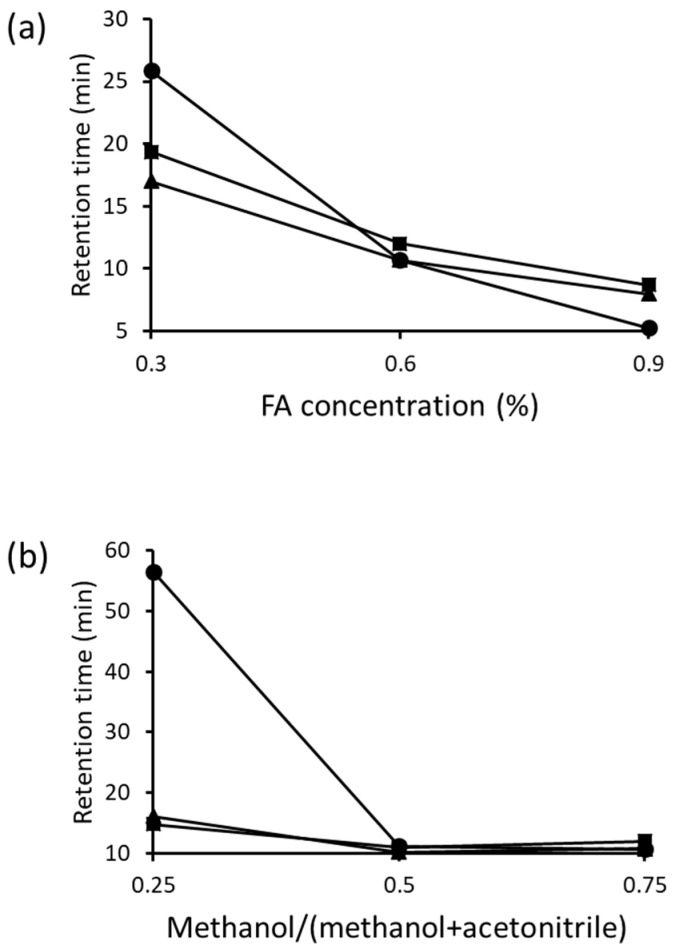
Effects of (**a**) formate (FA) concentrations and (**b**) methanol contents in mobile phase on the retention times of NBD- ornithine (Orn) (■), NBD-citrulline (Cit) (▲), and NBD-argininosuccinate (ASA) (●). Column: InertSustain NH_2_ (2.1 mm × 150 mm, 5 μm, GL Sciences). Mobile phase: (**a**) FA in methanol–acetonitrile (75/25, *v/v*)), and (**b**) 0.6% FA in methanol–acetonitrile.

First, the FA concentration in the mobile phase (methanol–acetonitrile, 75/25) was varied. With increasing FA concentrations, the retention times of NBD-Orn, NBD-Cit, and NBD-ASA were significantly decreased (Figure 3a), which suggested the involvement of an anion exchange phenomenon during the elution. Next, the methanol content in the mobile phase (0.6% FA in methanol-acetonitrile) was varied. As shown in Figure 3b, changing the methanol content changed the retention time of the three NBD-amino acids. These results confirmed that NBD-Orn, NBD-Cit, and NBD-ASA were retained by a mixed mechanism (anion exchange and hydrophilic interaction).

NBD-Arg was not sufficiently retained on the amino column under any of the conditions examined. This is expected as Arg is a basic amino acid, and NBD-Arg has no negative charge in an acidic solution. Hence, a sulfonic acid column was employed to separate NBD-Arg. Mixed solutions of methanol–acetonitrile containing ammonium formate were used as the mobile phase. With decreasing ammonium formate and methanol contents, the retention time of NBD-Arg increased.

Thus, NBD-Orn, NBD-Cit, and NBD-ASA can be well retained on the amino column, while NBD-Arg can be retained on the sulfonic acid column.

### 2.3. 2D-LC Separation of NBD-Urea Cycle Amino Acids in Human Plasma

Under the optimised conditions, the urea cycle amino acids in human plasma samples were analysed with the 2D-LC method. 2D-LC can be performed in either on-line or off-line mode. The on-line mode is automated and has good repeatability, which are advantageous. However, it requires more complex instrumentation with an interface, and fraction collection is not flexible. Although the experiment in off-line mode is laborious, it suffers no such limitations. Hence, the off-line mode was used in this study.

**Figure 4:**
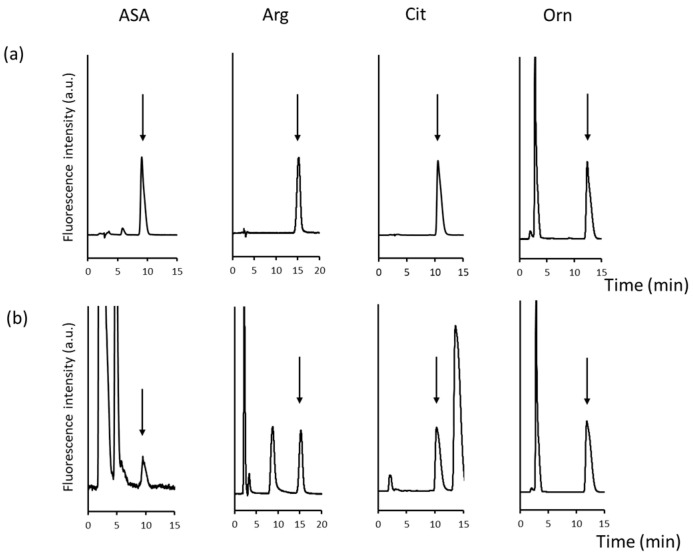
Analysis of NBD-argininosuccinate (ASA), NBD-arginine (Arg), NBD-citrulline (Cit), and NBD-ornithine (Orn). (**a**) In standard solution (each 150 nM) and (**b**) in human plasma in the second dimension. Arrow indicates the corresponding peak. Column: InertSustain NH_2_ (2.1 mm × 150 mm, 5 μm, GL Sciences) for NBD-Orn, -Cit, and -ASA, Inertsil CX (2.1 mm × 150 mm, 5 μm, GL Sciences) for NBD-Arg. Mobile phase: methanol–acetonitrile–FA (75/25/0.6, *v/v/v*) for NBD-Orn, -Cit, and -ASA, and 5 mM ammonium formate in methanol–acetonitrile (90/10, *v/v*) for NBD-Arg.

Urea cycle amino acids were first derivatised with NBD-F and then separated on an ODS column. Each peak of NBD-urea cycle amino acid was collected as a fraction, and the entire peak was collected for quantification. In total, 100 μL was collected, which corresponded to 1 min. From these, 30 μL NBD-Orn, NBD-Cit, and NBD-ASA and 10 μL NBD-Arg were introduced into the second dimension. The chromatographic conditions are described in the experimental section, which were chosen as each NBD-amino acid was retained for 10–15 min. Single peaks were obtained for the amino acids (Figure 4a). In the first dimension, NBD-Cit was co-eluted with NBD-Ser, and NBD-Arg and NBD-Asn were not separated well. Both the peaks were baseline separated in the second dimension.

Further, human plasma samples were analysed to check the interference from the endogenous substances in the matrix of real biological samples. Figure 2b and Figure 4b show chromatograms obtained from the first- and second-dimension analyses of human plasma, respectively. In the second dimension, there were several peaks that were not observed in the analysis of the standards, although such peaks did not interfere with the detection of the NBD-urea cycle amino acids. This result indicated that the 2D-LC system could be effectively used to analyse the urea cycle amino acids. 

The concentrations of urea cycle amino acids in human plasma were calculated. Arg, Orn, Cit, and ASA concentrations in human plasma were 30.2 ± 2.2, 101 ± 9.5, 22.3 ± 0.96, and 0.84 ± 0.07 μM, respectively. For Arg, Orn, and Cit, the obtained values are similar to the previously reported values [31,32,33]. To the best of our knowledge, ASA concentration in human plasma has not been reported using validated methods, and this is the first study to report the ASA concentration in human plasma.

### 2.4. Method Validation

The data validation for the developed method is shown in Table 1. The LOD and LOQ of NBD-Arg were higher than those of the other three NBD-amino acids because the injection volume of NBD-Arg in the second dimension was lower compared to the other NBD-amino acids. The calibration curves for each NBD-urea cycle amino acid showed good linearity, with correlation coefficients higher than 0.9969. The intraday and interday precisions were less than 7.9% and 15%, respectively. Although the values are slightly high, probably because of the manual transfer of the fractions from the first dimension to the second dimension, they are in the acceptable range. This suggests that the proposed method is appropriate for a routine assay of urea cycle amino acids in human plasma.

## 3. Materials and Methods

### 3.1. Chemicals

Arginine (Arg), ornithine (Orn), citrulline (Cit), type-H amino acids standard solution, trifluoroacetic acid (TFA), ammonium formate, and formic acid (FA) were purchased from FUJIFILM Wako Pure Chemical (Osaka, Japan). The fluorescent derivatization agent, 4-fluoro-7-nitro-2,1,3-benzoxadiazole (NBD-F), was sourced from Dojindo Laboratories (Kumamoto, Japan). Argininosuccinate (ASA), asparagine, and human plasma were purchased from Sigma-Aldrich (St. Louis, MO, USA). Glutamine was obtained from Kyowa Hakko Kogyo (Tokyo, Japan). Acetonitrile and methanol (HPLC grade) were procured from Merck (Darmstadt, Germany). A Milli-Q system (Merck) was used to purify water. All the other chemicals were of reagent grade.

### 3.2. Pre-Treatment of Human Plasma Samples

In the initial stage of the sample preparation protocol, a precisely measured 100 μL aliquot of human plasma was meticulously combined with an equivalent volume of deionized water. Subsequently, a controlled introduction of 400 μL of methanol, along with an additional 400 μL of acetonitrile, was executed. The resulting concoction, comprising human plasma, water, methanol, and acetonitrile, underwent a centrifugation at 3000× *g* for 5 min at a refrigerated temperature of 4 °C. This centrifugal force, applied with precision, acted as a discriminating force, effecting the stratification of heavier proteinaceous constituents, thus facilitating the selective isolation of the supernatant. The extracted supernatant represents a refined fraction amenable to downstream analytical pursuits. The resulting analyte-enriched fraction is poised for subsequent scientific interrogations, enabling precise and reliable analytical outcomes untainted by the presence of proteins.

### 3.3. Fluorescence Derivatisation

The supernatants (100 μL) derived from human plasma samples underwent a systematic dehydration process, employing reduced pressure at 60 °C until complete dryness was achieved. Following this, a specific volume (10 μL) of either the residue or an amino acid standard solution was subjected to the addition of 90 or 80 μL of 0.4 M borate buffer (pH 8.5), depending on the nature of the sample. Subsequently, 10 μL of a meticulously prepared 20 mM NBD-F solution in acetonitrile was introduced into the reaction mixture, initiating the derivatization process. Controlled heating at 60 °C for a duration of 5 min ensured the completion of the derivatization reaction. Subsequent to the reaction, the mixture was rapidly cooled in an ice water bath, and the addition of 100 μL of 0.1 M HCl aqueous solution followed. The final prepared sample, comprising 10 μL of the reaction mixture, was precisely injected into the 2D-LC system, representing a pivotal step in the intricate analytical workflow.

### 3.4. Apparatus and Chromatographic Setup of 2D-LC

For the first dimension, a sophisticated Shimadzu (Kyoto, Japan) LC system was employed. The comprehensive system consisted of a DGU-20A degasser, two LC-20AD pumps, an SIL-30AC auto sampler, a CTO-20A column oven, an RF-20A fluorescence detector, and a CBM-20A system controller. LabSolutions software (ver. 5.111) facilitated the analysis of chromatograms, providing a user-friendly platform for data interpretation and processing. The fluorescence excitation and emission wavelengths were set at 470 and 530 nm, respectively. The separation was performed using an Inertsil ODS-4 column (1.5 mm × 250 mm, 5 μm, GL Sciences, Tokyo, Japan) with the column temperature carefully maintained at 40 °C to optimise performance. The optimised mobile phases were (A) water–acetonitrile–TFA (80/20/0.02, *v/v/v*) and (B) water–acetonitrile–TFA (20/80/0.02, *v/v/v*). The sample was eluted stepwise as follows: 0–15 min: 0% B, 15.01–35 min: 40% B, 35.01–45 min: 100% B. The flow rate was consistently maintained at 0.1 mL/min, ensuring a steady and controlled progression of the elution process.

For the second dimension, an HPLC system (Jasco, Tokyo, Japan) equipped with a DG-980-50 3-line degasser, an LG-2080-02 ternary gradient unit, a PU-2080 pump, an AS-950 auto sampler, a CO-965 column oven, and an FP-920S fluorescence detector was used. The chromatograms were analysed using Chromato-Pro software (Run Time Corporation, Tokyo, Japan). Fluorescence signals were detected at 530 nm with an excitation wavelength of 470 nm. The analytical column selected for NBD-Orn, -Cit, and -ASA was InertSustain NH_2_ (2.1 mm × 150 mm, 5 μm, GL Sciences), and Inertsil CX (2.1 mm × 150 mm, 5 μm, GL Sciences) was chosen for NBD-Arg. Both the columns were maintained at a temperature of 40 °C, and the flow rate was 0.2 mL/min. The mobile phase composition for InertSustain NH_2_ consisted of methanol–acetonitrile–FA (75/25/0.6, *v/v/v*), and the injection volume was 30 μL. On the other hand, the mobile phase for Inertsil CX was composed of 5 mM ammonium formate in methanol–acetonitrile (90/10, *v/v*), and the injection volume was 10 μL.

### 3.5. Method Validation

In order to validate the efficacy of the developed 2D-LC method, a comprehensive assessment was conducted, encompassing calibration curves, limit of detection (LOD), limit of quantification (LOQ), precision, and accuracy, collectively shaping a comprehensive understanding of the method’s analytical performance. The calibration curves, pivotal in establishing a quantitative foundation, were meticulously constructed. Standard samples, spanning a spectrum of concentrations for urea cycle amino acids (Arg, Orn, Cit: 15–600 nM ASA: 3–600 nM), were judiciously prepared to span the dynamic range of interest. The relationship between the concentrations of these amino acids and their corresponding peak areas, derived from NBD derivatization, was intricately elucidated. Employing a robust least-squares regression analysis, key parameters such as slope, intercept, and correlation coefficient were meticulously extracted. In the realm of sensitivity analysis, the LOD and LOQ assumed paramount importance. These pivotal parameters were meticulously determined at signal-to-noise ratios (S/N) of 3 and 10, respectively, providing critical insights into the method’s sensitivity. The precision of the developed method underwent a rigorous and systematic evaluation, focusing on its performance in the analysis of human plasma samples. To ascertain the intricacies of precision, a meticulous approach was adopted, involving five replicate measurements conducted on the same day, thereby providing insights into intraday precision. This granular examination was complemented by a comprehensive assessment of interday precision, extending over five successive days. By scrutinizing the method’s reproducibility over varying time scales, a nuanced understanding of its precision dynamics was achieved. In addition to precision, the recovery of urea cycle amino acids was a focal point of investigation. This critical parameter was meticulously calculated through a comparative analysis. The slopes derived from human plasma samples, strategically spiked with standard amino acids, were juxtaposed against those obtained from standard amino acid samples. This comparative approach not only gauged the accuracy of the method but also shed light on its proficiency in quantifying urea cycle amino acids within the complex milieu of human plasma. The incorporation of such detailed recovery assessments ensures a robust characterization of the method’s analytical performance, further bolstering its applicability in the quantification of amino acids within the intricate matrix of biological samples.

## 4. Conclusions

An off-line 2D-LC system was developed and employed for the quantification of urea cycle amino acids (Arg, Orn, Cit, and ASA) in human plasma. After the fluorescence derivatisation of urea cycle amino acids with NBD-F, 2D separation was performed. In the first dimension, NBD-urea cycle amino acids were completely separated from each other on a reversed-phase column. A fraction of each NBD-urea cycle amino acid was transferred to ion-exchange columns, and all the NBD-urea cycle amino acids were separated from the other amino acids and endogenous interfering peaks. Owing to the high separation efficiency of the 2D-LC system, all the four amino acids in plasma were quantified, and the method was successfully validated. Analysis by this developed method can offer new insights into the physiological roles of urea cycle, especially in some diseases.

## Figures and Tables

**Table 1:** Validation data of the developed method for the analysis of human plasma samples.

	Limit of Detection [nM]	Limit of Quantification [nM]	Calibration Range [nM]	r^2^	Intraday Precision (%) (*n* = 5)	Interday Precision (%) (*n* = 4–5)	Recovery (%) (*n* = 3)
Arg	2.6	8.5	15–600	0.9985	4.0	3.8	95.4
Orn	0.96	3.2	15–600	0.9998	7.9	15	109
Cit	0.43	1.4	15–600	0.9988	0.98	7.7	99.9
ASA	0.53	1.8	3–600	0.9969	7.9	6.9	86.9

## Data Availability

Data are contained within the article.
